# A Morton's Neuroma Invading the Proper Digital Nerve

**DOI:** 10.7759/cureus.8920

**Published:** 2020-06-30

**Authors:** Steven R Edwards, Dean Samaras

**Affiliations:** 1 Surgery, Australasian College of Podiatric Surgeons, Melbourne, AUS; 2 Podiatry, La Trobe University, Bundoora, AUS; 3 Podiatric Surgery, Australasian College of Podiatric Surgeons, Melbourne, AUS

**Keywords:** neuroma, peripheral nerve surgeries, neurosurgery, foot tumours, orthopaedic disease

## Abstract

We report a case of a 55-year-old female with extreme right fourth toe pain of unknown origin that was resistant to conservative care. Resection confirmed invasion of the neuroma into the fourth digit with hypertrophy and herniation of the proper digital nerve. The patient experienced an uneventful recovery with some minor neurogenic symptoms experienced at six months postoperatively that resolved with off-loading padding and heat massage. Complete pain relief was reported at her 12-month review. Isolated neuroma within a toe results in digital pain that may respond to excision.

## Introduction

Neuromas of the forefoot are common conditions presenting to outpatient clinics and are generally amenable to conservative care [1-3]. Of these, Morton’s neuroma, the most common variant, was first described by Civinini in 1835 and refers to the nerve within the third intermetatarsal space [1].

There is a lack of epidemiological data regarding forefoot neuromas, and their prevalence is unknown [2]. They appear more common in females (75%-95%), and the average age of patients is 50 years [2,4,5]. High-arch feet and regularly wearing tight-fitting shoes appear to be risk factors [6].

Digital neuromas, neuromas affecting the proper digital nerve of one toe, may have a similar morphology, but to our knowledge there is no literature on their prevalence or treatment. Herein, we discuss our treatment method of such a case. To our knowledge, this is the first reported case of a proper digital neuroma within a toe.

## Case presentation

A 55-year-old female was referred reporting 12 months of extreme right fourth toe pain. She was of generally good health, taking 600 mg of lithium twice daily for bipolar disorder. She was a current smoker, averaging 10 cigarettes daily. Conservative treatments over the preceding 12 months had failed to reduce her symptoms. This included two corticosteroid injections using a betamethasone sodium acetate/lignocaine hydrochloride mix, one pair of non-prescription orthotics, physical therapy with heat massage, and several prescriptions of opioid-based analgesics.

**Figure 1:**
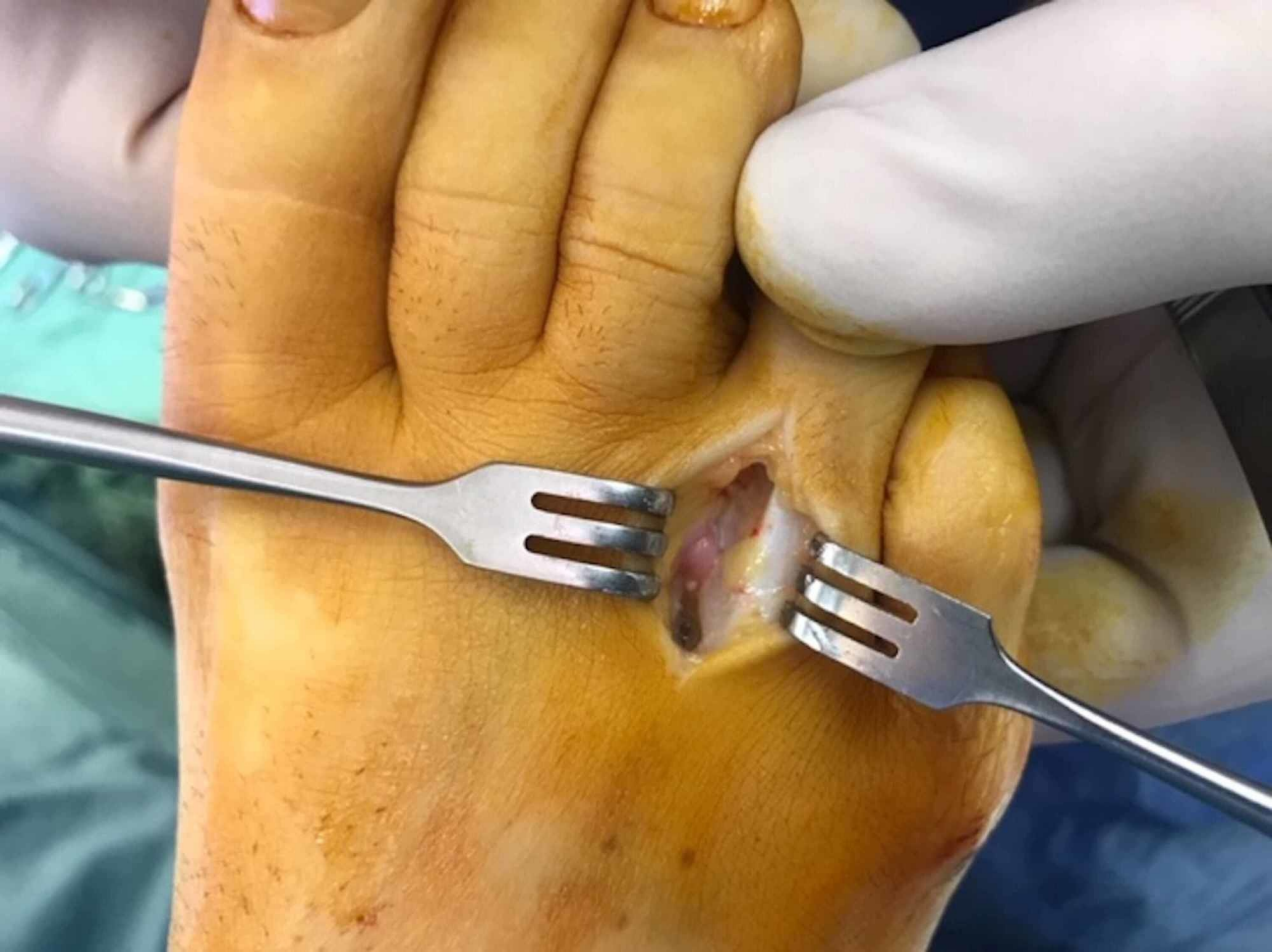
The proper digital nerve was resected via a dorsal approach to reduce the risk of plantar scarring.

**Figure 2:**
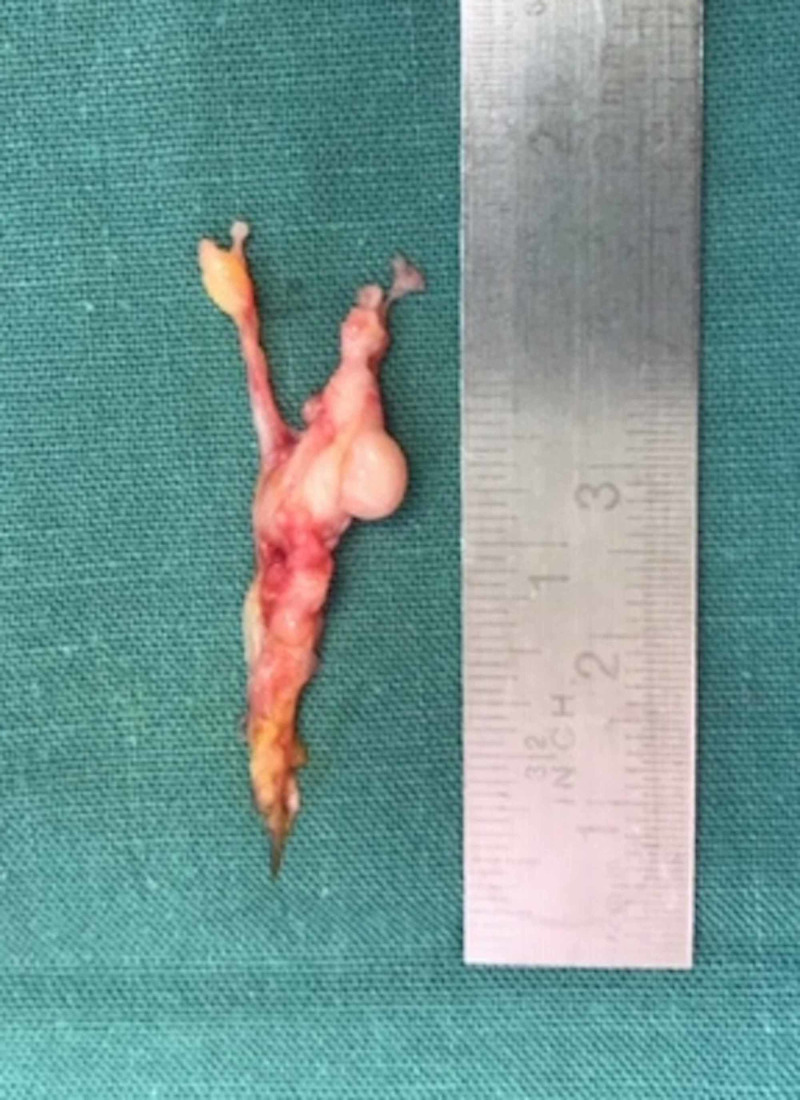
The resected nerve showing hypertrophy and herniation of the proper digital branch corresponding to the patient's focal area of pain.

Diagnostic ultrasound (US) showed a third webspace burial neuroma complex measuring 11 x 4.5 mm. Resection was performed via a dorsal approach (Figure 1). The specimen exhibited thickening and herniation of the proper digital branch of the fourth toe (Figure 2) which corresponded to her isolated site of pain. At six months, she experienced some mild neurogenic symptoms that were abated with off-loading padding and heat massage.

## Discussion

Neuromas of the forefoot often present with intermittent lancing pain, burning and numbness of adjacent toes, or a decrease in sensation in the area of the nerve [7]. There is controversy in the literature regarding their cause, with four main theories existing [4]. First, chronic trauma, where stress from the adjacent forefoot structures causes repetitive trauma to the nerve [4]. Second, ischaemia, whereby neurofibrosis occurs from reduced blood flow to the nerve via abnormalities to the feeder artery [8,9]. Third, intermetatarsal bursitis, where there is adherence between the nerve and the bursa [10-14]. Fourth, entrapment, where the nerve becomes entrapped between the plantar aspect of the foot and the transverse intermetatarsal ligament [15].

Although their cause is controversial, the pathophysiology of forefoot neuromas remains constant. Histological studies show perineurial and epineural fibrosis, degenerative vascular changes, arrested axonal nerve endings, demyelination of the axon, fibrinoid degeneration, and an increase in sympathetic nerve fibres [8]. 

Neuromas invading the proper digital nerve have been previously reported in the literature but only of the fifth proper digital nerve being encountered during the correction of bunionette deformities [16]. Many anatomy texts and illustrations describe the proper digital nerve of the fifth toe as being normally enlarged and convoluted as it passes the neck and condyles of the fifth metatarsal. This cause does not appear to be the case with digital neuromas of the fourth digit.

We report a single case report of a proper digital nerve of the fourth toe that resulted in extreme pain. This appears to be the first reported case of a proper digital nerve not involving the fifth toe. 

## Conclusions

This appears to be the first case in the literature of extreme isolated toe pain caused by a neuroma within the digit. Surgical resection was performed which resolved the patient's symptoms, albeit with some mild neurogenic pain arising at six months postoperatively that were abated with off-loading padding and heat massage. Surgical resection in a case of proper digital neuroma appears to provide effective pain relief and patient satisfaction.
